# In Vitro Metabolism of a Benzofuran-Substituted Nitazene: Ethyleneoxynitazene

**DOI:** 10.3390/metabo15100679

**Published:** 2025-10-21

**Authors:** Omayema Taoussi, Duygu Yeşim Ovat, Francesco Tavoletta, Anastasio Tini, Giulia Bambagiotti, Jeremy Carlier, Volker Auwärter, Francesco Paolo Busardò, Diletta Berardinelli

**Affiliations:** 1 Section of Legal Medicine, Department of Biomedical Sciences and Public Health, Marche Polytechnic University, 60126 Ancona, Italy; 2 Toxicology and Pharmaceutical Science, Institute on Drug Abuse, Ege University, Bornova 35040, Turkey; yesim.karabulut90@gmail.com; 3 Institute of Forensic Medicine, Medical Center-University of Freiburg, Faculty of Medicine, University of Freiburg, Albertstraße 9, 79104 Freiburg, Germany; volker.auwaerter@uniklinik-freiburg.de

**Keywords:** ethyleneoxynitazene, nitazene, benzylbenzimidazole opioid, new synthetic opioid, new psychoactive substance, metabolism, metabolomics, high-resolution mass spectrometry, biomarker

## Abstract

**Background/Objectives**: New synthetic opioids (NSOs) like nitazenes pose significant public health risks due to their high potency and increasing prevalence. Ethyleneoxynitazene, a benzofuran-containing nitazene, recently emerged on the illicit market and was identified in seizures in Europe. Although no intoxications have been reported to date, its µ-opioid receptor activity raises concern. This study investigated the metabolism of ethyleneoxynitazene to better understand its pharmacological profile, toxicity, and detectability in clinical and forensic contexts. **Methods**: Ethyleneoxynitazene was incubated with cryopreserved human hepatocytes pooled from 10 donors. Metabolites were detected by liquid chromatography coupled with high-resolution tandem mass spectrometry (LC-HRMS/MS) and identified using Compound Discoverer (Thermo Scientific; Waltham, MA, USA); detection and identification were assisted by in silico metabolite predictions with BioTransformer. **Results**: Sixteen metabolites were identified, with major biotransformations including *N*-deethylation at the *N*,*N*-diethylethanamine chain, hydroxylation at the dihydrofuran ring, and dihydrofuran ring opening via oxidative cleavage, leading to the formation of the corresponding ethanoic acid. **Conclusions**: This study provides the first characterization of the metabolism of a nitazene without an alkoxyphenyl moiety; the absence of this particular group reflects significant differences in the pharmacokinetic and pharmacodynamic profile compared to other nitazenes. We propose *N*-deethyl-3′-ethanoic acid-4′-hydroxy ethyleneoxynitazene, *N*-deethyl-hydroxy ethyleneoxynitazene, 3′-ethanoic acid-4′-hydroxy ethyleneoxynitazene, hydroxy ethyleneoxynitazene, and *N*-deethyl ethyleneoxynitazene as metabolite biomarkers of ethyleneoxynitazene consumption in clinical and forensic toxicology. Given the potential activity of some metabolites and interindividual variability in metabolic pathways, further studies are warranted to refine these findings through the analysis of biological samples from multiple ethyleneoxynitazene-positive cases.

## 1. Introduction

New synthetic opioids (NSOs) are substances with heroin-like effects and pose growing challenges to public health and law enforcement agencies. In the early 2010s, the United States have experienced an unprecedented increase in overdose fatalities with the emergence of illicitly produced fentanyl and NSOs on the recreational drug market. The “opioid crisis” reshaped the demographics of opioid-related deaths, traditionally associated with heroin and methadone use, and resulted in the death of nearly 500,000 people from 1996 to 2019; overdose deaths even contributed to the decrease in life expectancy for US Americans during 2014 to 2017 [1,2]. This trend has since spread globally, particularly in North America, Oceania as well as Western and Central Europe [3,4]. Following the scheduling of fentanyl structural analogs in 2019 in China and the United States, users turned to alternative NSOs, further worsening the crisis [5,6,7]. Among those alternative NSOs, nitazenes (also known as 2-benzylbenzimidazole opioids), have dominated the market due to their high potency at the µ-opioid receptor in the central nervous system (their activity at the δ- and κ-opioid receptors being marginal) [8], causing severe adverse effects, including respiratory depression and death [7,9,10].

**Figure 1:**
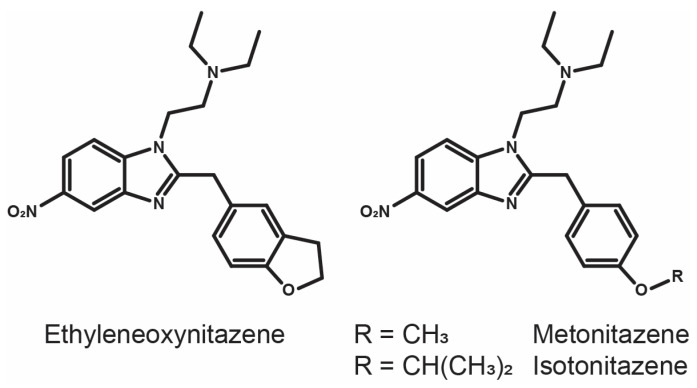
Chemical structure of ethyleneoxynitazene and two analogs.

Ethyleneoxynitazene (2-{2-[(2,3-dihydro-1-benzofuran-5-yl)methyl]-5-nitro-1*H*-benzimidazol-1-yl}-*N*,*N*-diethylethan-1-amine) has surfaced the illicit drug market in 2023 in Estonia, where it was identified in a powder seized by the Central Criminal Police [11]. Ethyleneoxynitazene is a structural analog of potent *N*,*N*-diethyletamine nitazenes such as isotonitazene and metonitazene, which were developed in the 1950s and were predominant on the NSO market in 2019–2020 [7,12,13], with a benzofuran moiety instead of an alkoxybenzene (Figure 1). No intoxication cases have been reported to date. However, experimental data have shown that ethyleneoxynitazene acts as a full agonist at the µ-opioid receptor, with an affinity of 57.9 nmol/L (radioligand binding assay) and a potency of 70.0 nmol/L (*β*-arrestin2 recruitment assay) [14]. The same authors also reported moderate antinociceptive effects and reduced locomotor activity in rats, categorizing the substance as a moderate-activity opioid [14].

Studying the metabolism of ethyleneoxynitazene is essential to better understand its pharmacological profile and potential toxicity. Considering its activity at the µ-opioid receptor, identifying its metabolic pathways can help to understand whether metabolites contribute to its overall effect, influence its duration of action, or pose additional toxicological risks. Indeed, previous studies have shown that nitazene metabolites are often pharmacologically active [15,16] and their metabolism involves highly polymorphic enzymes [17]. Understanding ethyleneoxynitazene metabolism can also help improve detectability in forensic or clinical settings, to document consumption and interpret drug-related intoxication cases and fatalities [18,19]. Various studies have assessed the metabolism of structural analogs [16,18,19,20,21,22], but there is currently no data available on the metabolism of ethyleneoxynitazene. The present study investigated ethyleneoxynitazene metabolism in humans using 10-donor-pooled human hepatocyte incubations, liquid chromatography-high-resolution tandem mass spectrometry (LC-HRMS/MS) screening, and software-assisted data mining.

## 2. Materials and Methods

### 2.1. Chemicals and Reagents

LC-MS grade acetonitrile, water, methanol, and formic acid were obtained from Carlo Erba (Cornaredo, Italy). Ethyleneoxynitazene and diclofenac analytical standards were obtained from Cayman Chemical (Ann Arbor, MI, USA) and Sigma Aldrich (Milan, Italy), respectively. Stock solutions of standards were prepared at a concentration of 1 mg/mL in methanol and stored at −20 °C. Sigma Aldrich supplied Williams’ Medium E, HEPES buffer (2-[4-(2-hydroxyethyl)-1-piperazinyl]ethanesulfonic acid) and *l*-glutamine. Supplemented Williams’ medium E (sWME) containing HEPES (2 mmol/L) and L-glutamine (20 mmol/L) was prepared extemporaneously. Human hepatocytes cryopreserved from 10 donors, thawing medium, and 0.4% trypan blue were obtained from Lonza (Basel, Switzerland).

### 2.2. In Silico Metabolite Predictions

Metabolites of ethyleneoxynitazene in humans were predicted in silico using the BioTransformer (v. 3.0) freeware [22,23]. The metabolite list was generated using the simplified molecular-input line-entry system (SMILES) identification of the molecule, as obtained with ChemSketch (ACD/ChemSketch, for Microsoft Windows, v. 2020. 1. 2). The option for prediction of metabolic transformation was “Human and Human Gut Microbial Transformation (AllHuman)” and the option for CYP450 Mode was “Combined”. The predicted metabolites were used for the LC-HRMS/MS inclusion lists and the data mining biotransformation list.

### 2.3. Hepatocyte Incubation

The experimental design for the human hepatocyte incubations followed our previously described protocol [24]. Hepatocytes were thawed in a proprietary thawing solution at 37 °C, then resuspended in sWME to a concentration of 2 × 10^6^ viable cells/mL (viability assessed using the trypan blue exclusion method). 250 μL of hepatocytes suspension was gently mixed with 250 μL of 20 μmol/L ethyleneoxynitazene in sWME at 37 °C in a 24-well culture dishes, alongside with positive and negative controls (diclofenac and hepatocyte suspension in sWME; hepatocyte suspension in sWME without additional substance; ethyleneoxynitazene in sWME without hepatocytes). The well plates were incubated at 37 °C for 0 or 3 h. The reactions were quenched by adding 250 µL of ice-cold acetonitrile and centrifugating the mixture at 15,000× *g* for 10 min, and the incubates were stored in polypropylene microtubes at −80 °C.

### 2.4. Sample Preparation

Thawed incubates were vortex mixed and centrifuged at 15,000× *g* for 10 min at room temperature. 100 μL supernatant was mixed with 100 μL acetonitrile and re-centrifuged under the same conditions. The supernatants were evaporated to dryness under a nitrogen stream at 37 °C, and the dry residues were reconstituted in 100 μL water:acetonitrile (90:10, *v*/*v*) with 0.1% formic acid. After centrifugation under the same conditions, the supernatants were transferred to LC glass vials.

### 2.5. LC-HRMS/MS Analysis

LC-HRMS/MS analysis was performed using DIONEX UltiMate 3000 LC coupled with a Q-Exactive quadrupole-Orbitrap hybrid HRMS with a heated-electrospray-ionization (HESI) source (Thermo Scientific; Waltham, MA, USA).

#### 2.5.1. LC Conditions

The volume of injection was 10 µL. Chromatographic separation was performed using a Kinetex^®^ Biphenyl column (150 × 2.1 mm, 2.6 μm; Phenomenex, Torrance, CA, USA. The mobile phases (MPs) were 0.1% formic acid in water (MPA) and 0.1% formic acid in acetonitrile (MPB), and ran through the column at a 0.4 mL/min flow rate at 37 °C. Gradient elution started at 5% MPB, held for 2 min, followed by a linear increase to 25% over 14 min, then to 50% in 5 min, and to 95% in 1 min, held for 5 min. Initial conditions (5% MPB) were restored in 0.1 min and maintained until the end of the 30 min run.

#### 2.5.2. HRMS/MS Conditions

Prior the analysis, the ionization source settings were optimized infusing ethyleneoxynitazene standard at 1 μg/mL in MPA:MPB (90:10, *v*/*v*) at a 5 µg/min flow rate. Experiments were carried out using both positive- and negative-ionization modes. HESI conditions were: 50 au for the sheath gas flow rate, 10 au for the auxiliary gas flow rate, ±3.5 kV for the spray voltage, 300 °C for the capillary and auxiliary gas heater temperatures, and 50 for the S-lens radio frequency; there was no use of sweep gas. The orbitrap was calibrated before analysis, and mass deviation was adjusted at each injection using a lock mass list.

Data were acquired between 1 and 25 min of the LC gradient in full-scan HRMS (FullMS) and data-dependent MS/MS (ddMS^2^) modes. The FullMS acquisition range was *m*/*z* 250–700 with a resolution of 70,000 (full width at half maximum, FWHM at *m*/*z* 200); the automatic gain control (AGC) target was set to 3 × 10^6^ with a maximum injection time (IT) of 200 ms. A maximum of five ddMS^2^ scans were performed per FullMS scan with a dynamic exclusion of 2.0 s and an intensity threshold of 10^4^. The ddMS^2^ isolation window was *m*/*z* 1.2 with a resolution of 17,500; normalized collision energy (NCE) values were 40, 55, 80 au; the AGC target was set to 2 × 10^5^ with a maximum IT of 64 ms. The ddMS^2^ acquisition relied on an inclusion list (with the “select others if idle” option) derived from in silico metabolite prediction results (Supplementary Table S1).

### 2.6. Data Mining

The LC-HRMS data were processed using Compound Discoverer software (v. 3.1.1.12, Thermo Scientific) following a semi-automated workflow previously implemented with minor modifications [16,25]. Briefly, ions detected in the HRMS data were compared against a theoretical metabolite list, which was generated based on predicted Phase I and Phase II metabolic transformations (Supplementary Table S2). The data were filtered using an intensity threshold of 5 × 10^3^ and a mass accuracy of 5 ppm. Detected ions were further matched with mzCloud libraries, including Counterfeit Drugs (Therapeutic), Drugs of Abuse/Illegal Drugs, and Therapeutics/Prescription Drugs, as well as the ChemSpider-Cayman Chemical and DrugBank databases. For this comparison, the intensity threshold was set to 10^5^, with a mass tolerance of 5 ppm for HRMS and 10 ppm for HRMS/MS. Chromatographic peaks exhibiting intensities equal to or higher than the control samples were flagged for further manual examination.

## 3. Results

### 3.1. In Silico Metabolite Predictions

BioTransformer predicted 11 primary metabolites (pA1-pA11) and 60 secondary metabolites (pAX-1-pAX-n, where pAX is the corresponding primary metabolite and *n* ≤ 12). The in silico metabolite prediction results are provided in Supplementary Table S3. Hydroxylation and subsequent dehydrogenation to carbonyl, *N*-oxidation at the diethylamine side chain, *N*-dealkylation at the diethylamine side chain, and nitro reduction were prominent Phase I reactions. In addition, phase II conjugations identified included *O*-glucuronidation or *O*-sulfation subsequent to hydroxylation/oxidation, and *N*-glucuronidation at the diethylamine side chain. The predicted metabolites were used to build the ddMS^2^ inclusion list (Section 2.5.2), and all their transformations were incorporated into a list of potential reactions for automated data mining (Section 2.6).

### 3.2. Ethyleneoxynitazene LC-HRMS/MS Fragmentation

**Figure 2:**
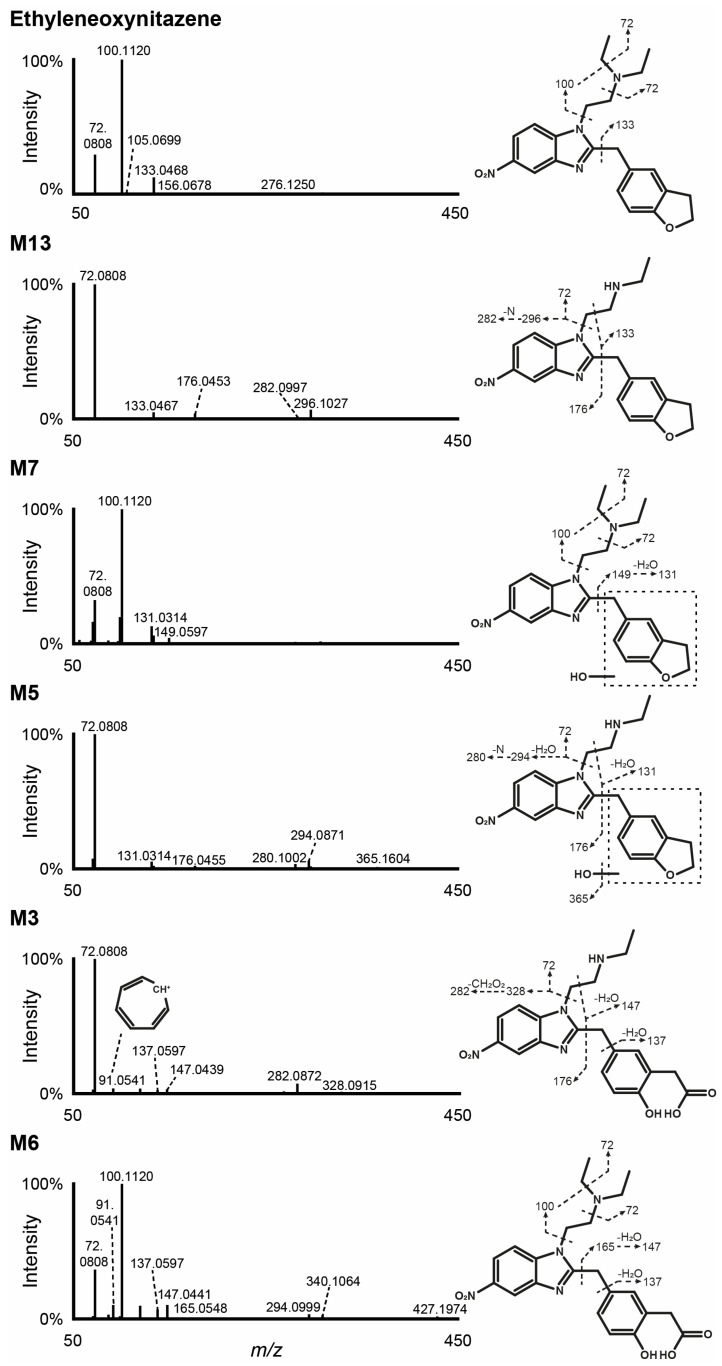
High-resolution tandem mass spectrometry spectra after positive-electrospray ionization and suggested fragmentation of ethyleneoxynitazene and metabolites. Screenshots of the spectra as they appear in the LC-HRMS/MS data processing software FreeStyle (v.1.6, Thermo Scientific) are provided in Supplementary Figure S1.

Ethyleneoxynitazene was detected only in positive ionization mode under the current analytical conditions, and yielded only few fragments, consistent with structural analogs [16,20] (Figure 2). The molecule eluted at 15.16 min during the chromatographic run, and showed a signal at *m*/*z* 395.2073, corresponding to the protonated ion [M+H]^+^. The mass spectrum displayed three main fragments, with the most prominent signals corresponding to the cleavage of the *N*,*N*-diethylethanamine (C_6_H_14_N^+^, *m*/*z* 100.1120) and diethylamine (C_4_H_10_N^+^, *m*/*z* 72.0808) groups from the parent, the nitrogen atom of the aliphatic amine being the main protonation site. The third fragment at *m*/*z* 133.0468 was the result of the cleavage of the (2,3-dihydro-1-benzofuran-5-yl)methyl moiety (C_9_H_9_O^+^).

Unless otherwise specified, all ions and MS/MS spectra described in the manuscript are in positive-ionization mode.

### 3.3. Metabolite Identification in Hepatocyte Incubations

**Figure 3:**
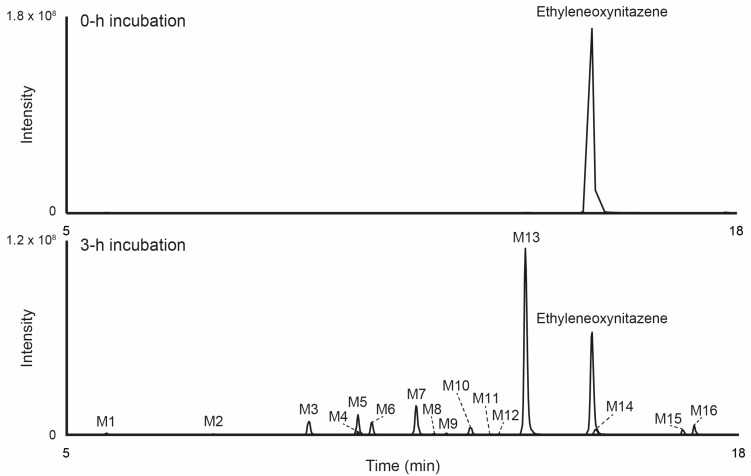
Extracted-ion chromatogram in positive-ionization mode of ethyleneoxynitazene and metabolites after ethyleneoxynitazene incubation with 10-donor-pooled human hepatocytes for 0 and 3 h. Mass tolerance, 5 ppm.

Following incubation with human hepatocytes, 16 metabolites were identified (designated as M1 to M16 based on increasing retention times). The LC-HRMS signal area for ethyleneoxynitazene decreased from 9.1 × 10^8^ at 0 h to 3.1 × 10^8^ after 3 h of incubation, indicating significant metabolization. The chromatogram showing the extracted-ion profiles of the parent compound and its metabolites after 3 h of incubation is provided in Figure 3. The main metabolic transformations observed were *N*-deethylation, hydroxylation at the (2,3-dihydro-1-benzofuran-5-yl)methyl moiety, and oxidative cleavage of the dihydrofuran leading, either as single processes or in combination. Additional reactions included nitro reduction, oxidation, oxidative deamination subsequent to *N*,*N*-dideethylation, and Phase II *O*-glucuronidation and *N*-acetylation. Table 1 provides an overview of the elemental compositions, retention times, accurate molecular ion masses, and LC-HRMS peak areas for ethyleneoxynitazene and its metabolites after 3 h of incubation. The fragmentation patterns for the most intense ethyleneoxynitazene metabolites in positive-ionization mode are displayed in Figure 2. The structure elucidation process of the main metabolites is detailed in the following subsections.

#### 3.3.1. N-Deethylation

M13 was identified as the predominant metabolite, resulting from deethylation at the aliphatic amine. This transformation was evidenced by a mass shift of −28.0314 Da relative to the parent compound, corresponding to the loss of C_2_H_4_. The metabolite was observed at a retention time of 13.86 min in the LC gradient and exhibited a base peak at *m*/*z* 367.1759. The fragmentation pattern of M13 included some key fragments of the parent compound, specifically the base peak at *m*/*z* 72.0808 (C_4_H_10_N^+^) from the diethylamine side chain, and *m*/*z* 133.0467 (C_9_H_9_O^+^), indicating that the (2,3-dihydro-1-benzofuran-5-yl)methyl moiety remained unchanged. However, the absence of the ion at *m*/*z* 100.1121 ± 5 ppm, major fragment in the fragmentation pattern of ethyleneoxynitazene, points towards an *N*-deethylation at the *N*,*N*-diethylethanamine side chain. Additional minor fragments were also detected (C_8_H_6_N_3_O_2_^+^, *m*/*z* 176.0453; C_16_H_14_N_3_O_3_^+^, *m*/*z* 296.1027; and C_16_H_14_N_2_O_3_^+^, *m*/*z* 282.0997).

#### 3.3.2. Hydroxylation

M7 was produced through hydroxylation (+O), as indicated by a +15.9950 Da mass shift from the parent compound, and eluted at 11.76 min of the LC run with a signal at *m*/*z* 411.2023. M7 was the second most intense metabolite, although it was approximately 7 times less intense than M13. The mass spectrum of M7 displayed major fragments at *m*/*z* 72.0808 (C_4_H_10_N^+^) and 100.1120 (C_6_H_14_N^+^), also detected in ethyleneoxynitazene fragmentation pattern, indicating that the *N*,*N*-diethylethanamine group was unchanged. The fragment at *m*/*z* 149.0597 resulted from the cleavage of the (2,3-dihydro-1-benzofuran-5-yl)methyl moiety with the addition of one oxygen atom (C_9_H_9_O_2_^+^) and further yielded a fragment at *m*/*z* 131.0314 (C_9_H_7_O^+^) through loss of water. This suggests that the transformation likely occurred either at the dihydrofuran ring or at the methyl linker of the (2,3-dihydro-1-benzofuran-5-yl)methyl group.

#### 3.3.3. Combination of N-Deethylation and Hydroxylation

The combination of *N*-deethylation (−C_2_H_4_) at the *N*,*N*-diethylethanamine chain and hydroxylation (+O) at the dihydrofuran ring or at the methyl linker of the (2,3-dihydro-1-benzofuran-5-yl)methyl moiety occurred in M5. The metabolite eluted at 10.63 min, with a protonated ion at *m*/*z* 383.1712, i.e., a −12.0361 Da mass shift from ethyleneoxynitazene. As previously described, detection of the fragment at *m*/*z* 72.0808 (C_4_H_10_N^+^) and the absence of *m*/*z* 100.1121 ± 5 ppm (C_6_H_14_N^+^) supported the *N*-deethylation reaction, while the fragments at *m*/*z* 131.0314 (C_9_H_7_O^+^) and 176.0455 (C_8_H_6_N_3_O_2_^+^) substantiated the hydroxylation at the (2,3-dihydro-1-benzofuran-5-yl)methyl group.

#### 3.3.4. Combination of Hydroxylation and Oxidative Cleavage

M6 resulted from hydroxylation (+O) at the dihydrofuran ring and dihydrofuran ring opening via oxidative cleavage (+O), leading to the formation of the corresponding ethanoic acid, as shown by the +31.9897 Da mass shift from ethyleneoxynitazene (*m*/*z* 427.1970 at 10.91 min). The fragments of M6 at *m*/*z* 72.0808 (C_4_H_10_N^+^) and 100.1120 (C_6_H_14_N^+^) indicated that the aliphatic amine chain remained unaltered, as previously explained in previous subsections. However, the fragment at *m*/*z* 165.0548, corresponding to the cleavage of the (2,3-dihydro-1-benzofuran-5-yl)methyl moiety from the parent compound with the addition of two oxygen atoms (C_9_H_9_O_3_^+^), and the fragment at *m*/*z* 147.0441, resulting from subsequent water loss (C_9_H_7_O_2_^+^), suggested that all transformations occurred at the (2,3-dihydro-1-benzofuran-5-yl)methyl group. The presence of the tropylium ion (C_7_H_7_^+^) at *m*/*z* 91.0541, characteristic of phenyl rings, pointed towards the opening of the dihydrofuran group. M6 also produced a signal in negative-ionization mode, with a deprotonated ion at *m*/*z* 425.1841 and an intensity approximately 60% of that observed in positive-ionization mode, further supporting the formation of carboxylic acid prone to deprotonation.

#### 3.3.5. Combination of N-Deethylation, Hydroxylation, and Oxidative Cleavage

M3 was the result of all three main metabolic transformations, with the combination of *N*-deethylation (−C_2_H_4_) at the *N*,*N*-diethylethanamine chain, hydroxylation (+O) at the dihydrofuran ring, and dihydrofuran ring opening via oxidative cleavage (+O), leading to the formation of the corresponding ethanoic acid. These modifications were suggested by the +3.9588 Da mass shift from the parent compound (*m*/*z* 399.1661 at 9.68 min). As previously described, the presence of the fragment at *m*/*z* 72.0808 (C_4_H_10_N^+^) and the absence of *m*/*z* 100.1121 ± 5 ppm (C_6_H_14_N^+^) in the fragmentation spectrum of M3 indicated the *N*-deethylation reaction, while ions at *m*/*z* 91.0541 (C_7_H_7_^+^) and 147.0439 (C_9_H_7_O_2_^+^) were characteristic of the hydroxylation and oxidative opening of the dihydrofuran ring. An additional fragment at *m*/*z* 282.0872 (C_15_H_12_N_3_O_3_^+^), yielded via formic acid loss from *m*/*z* 328.0915 (C_16_H_14_N_3_O_5_^+^) after the loss of the *N*,*N*-diethylethanamine chain, further substantiated the presence of a carboxylic acid within the molecule. Additionally, as observed for M6, which also underwent oxidative cleavage/opening of the dihydrofuran ring, M3 produced a signal in negative-ionization mode (*m*/*z* 397.1530, ~60% of the intensity in positive-ionization mode), suggesting the formation of a carboxylic acid.

## 4. Discussion

### 4.1. Ethyleneoxynitazene Metabolic Pathway

**Figure 4:**
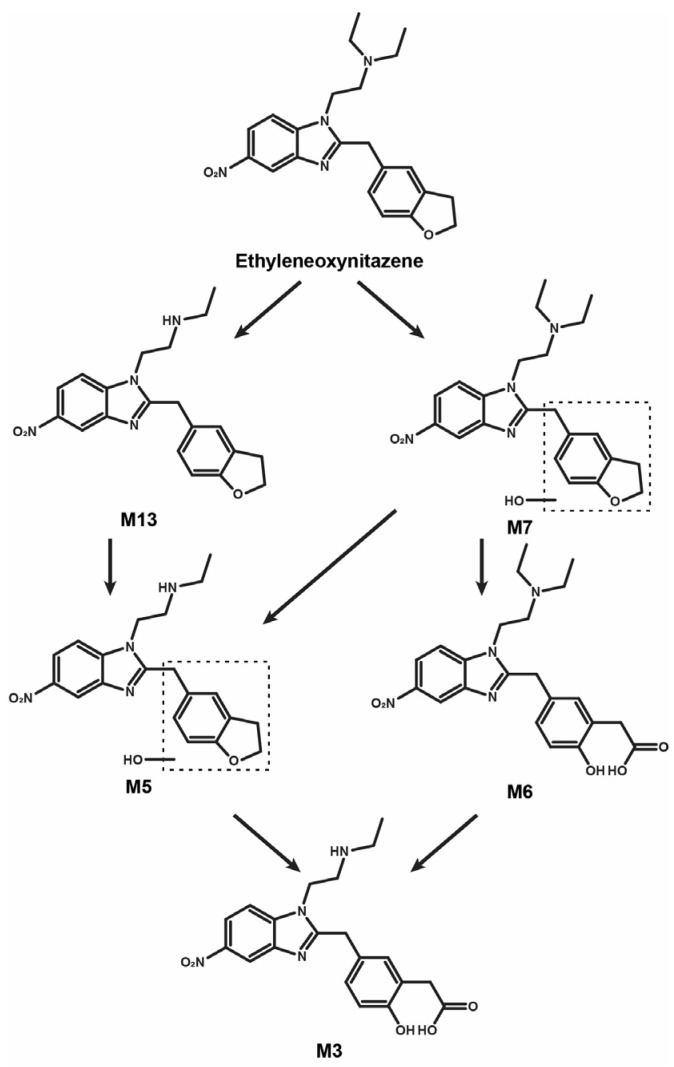
Proposed mechanism of metabolic transformations of ethyleneoxynitazene in humans (only main metabolites). Dashed box; uncertain position.

To the best of our knowledge, this is the first study to investigate the metabolism of a nitazene that does not bear an alkoxyphenyl substitution. This is an important observation, as previous studies have predominantly reported *O*-dealkylation at the alkoxyphenyl moiety as one of the main metabolic pathways for nitazenes [16,18,19,20,21,22], producing metabolites with marginal activity [9,15,26]. The lack of an alkoxyphenyl group might therefore have significant implications for the pharmacokinetics and pharmacodynamics of such compounds. Figure 4 displays the proposed human metabolic pathway of ethyleneoxynitazene. Three biotransformations were predominant: *N*-deethylation of the *N*,*N*-diethylethanamine chain, dihydrofuran hydroxylation, and dihydrofuran oxidative cleavage.

*N*-deethylation is a major pathway of nitazenes with an *N*,*N*-diethylethanamine side chain [16,18,19,21] and was the main transformation identified in the present study. *N*-deethylated metabolites are often predominant in blood in vivo but appear minor or are present in combination with *O*-dealkylation in urine, likely due to slower renal elimination. Interestingly, *N*-deethylated metabolites are also often pharmacologically active, and sometimes even more potent than the respective parent compounds [9,15,26]. For this reason, the presence of nitazenes with an *N*-ethylethanamine side chain has been increasingly reported on the illicit drug market in recent years [27]. The production of potentially active metabolites makes it essential to investigate their effects, to better understand their contribution to the overall pharmacokinetic and pharmacodynamic properties of the parent compound. However, the activity of *N*-deethyl ethyleneoxynitazene is currently unknown.

Oxidative cleavage of furan-containing compounds is a well-known metabolic transformation [28,29], although this is the first time it has been reported in the metabolism of a nitazene. In the case of ethyleneoxynitazene, oxidative cleavage is expected to yield an aldehyde (not detected), which is prone to subsequent carboxylation, as observed in the present study with the formation of ethanoic acid metabolites.

Another remarkable metabolic pathway that was minor in the present study is the reduction of the nitro group. Nitro reduction has been reported in previous studies as a major biotransformation for several nitazenes, yet its occurrence appears to exhibit significant interindividual variability and the reaction may be underestimated in human hepatocyte incubations [16,18,19]. This variability may be largely driven by the involvement of polymorphic enzymes, including those from the cytochrome P450 family, reductases, or other nitro-reducing enzymes [17], although extrahepatic metabolism should not be overlooked. Further investigation is needed to identify the specific enzymes responsible for the nitro reduction to better interpret nitazene-related intoxication cases and fatalities in analytical toxicology.

Lastly, M2 and M8 were the only Phase II metabolite identified in the incubates and displayed low intensity LC-HRMS peak area. The positive control with diclofenac, however, confirmed adequate metabolic activity throughout the experiments, including Phase II conjugation reactions. Interestingly, *O*-glucuronidation often is a major metabolic pathway of alkoxyphenyl-bearing nitazenes, following *O*-dealkylation, both in vitro with human hepatocyte incubations and in vivo [16,20]. The absence of such a moiety in the chemical structure of ethyleneoxynitazene explains the limited presence of Phase II metabolites observed in the present study, although *O*-glucuronidation or sulfation could hypothetically occur following hydroxylation or dihydrofuran oxidative cleavage.

### 4.2. In Silico Metabolite Predictions

Only 7 out of 16 metabolites identified in vitro were also potentially predicted in silico. Of note, the oxidative cleavage of the dihydrofuran ring and the oxidative deamination of the *N*,*N*-diethylamine side chain were not predicted. This shows that the model failed to accurately predict the metabolic fate of ethyleneoxynitazene, highlighting the crucial role of experimental investigations in metabolite identification. However, predictive approaches remain valuable for refining LC-HRMS/MS workflows and data mining, as seen in the present study.

### 4.3. Metabolite Biomarkers of Ethyleneoxynitazene Consumption

To the best of our knowledge, all metabolites identified in this study are specific to ethyleneoxynitazene consumption, as it is currently the only benzofuran-containing nitazene available on the illicit market. We propose M3 (*N*-deethyl-3′-ethanoic acid-4′-hydroxy ethyleneoxynitazene), M5 (*N*-deethyl-hydroxy ethyleneoxynitazene), M6 (3′-ethanoic acid-4′-hydroxy ethyleneoxynitazene), M7 (hydroxy ethyleneoxynitazene), and M13 (*N*-deethyl ethyleneoxynitazene) as metabolite biomarkers of ethyleneoxynitazene consumption in biological matrices. We do not recommend the hydrolysis of conjugated metabolites, as it would not significantly affect the detection capabilities of the main Phase I metabolites.

## 5. Conclusions

We investigated the in vitro hepatic metabolism of ethyleneoxynitazene, providing a solid foundation for future research on the pharmacokinetics and pharmacodynamics of this NSO, and identifying potential metabolite biomarkers of consumption for clinical and forensic applications.

Due to the potential over- or underestimation of certain nitazene metabolites and the polymorphic nature of the enzymes involved in their production, the analysis of biological samples from multiple positive cases is needed to further refine the present findings on ethyleneoxynitazene metabolism.

Additionally, since nitazene metabolites are often pharmacologically active, further studies should investigate the potency and efficacy of those identified in the present study to better interpret toxicological data in ethyleneoxynitazene-positive cases.

## Figures and Tables

**Table 1:** Metabolic transformation, elemental composition, retention time (RT), accurate mass of molecular ion, deviation from theoretical accurate mass, and liquid chromatography-high-resolution mass spectrometry peak area of ethyleneoxynitazene and metabolites in positive- and negative-ionization mode after 3 h Incubation with human hepatocytes. Mass tolerance, 5 ppm; ND, not detected.

ID	Biotransformation	Elemental Composition	RT, min	*m*/*z* [M+H]^+^ [M−H]^−^	Mass Error, (Δppm)	Peak Area in Incubates: [M+H]^+^ [M−H]^−^
M1	Nitro reduction	C_22_H_28_N_4_O	5.78	365.2343 ND	−1.95 -	3.6 × 10^6^ ND
M2	Nitro reduction + *N*-Acetylation	C_24_H_30_N_4_O_2_	7.83	407.2441 ND	−0.13 -	1.5 × 10^6^ ND
M3	*N*-Deethylation + Hydroxylation + Dihydrofuran opening to COOH	C_20_H_22_N_4_O_5_	9.68	399.1661 397.1530	−0.49 3.16	3.6 × 10^7^ 2.2 × 10^7^
M4	Hydration	C_22_H_28_N_4_O_4_	10.62	413.2179 411.2054	−1.04 3.94	6.5 × 10^6^ 2.1 × 10^6^
M5	*N*-Deethylation + Hydroxylation	C_20_H_22_N_4_O_4_	10.63	383.1712 ND	−0.47 -	4.8 × 10^7^ ND
M6	Hydroxylation + Dihydrofuran opening to COOH	C_22_H_26_N_4_O_5_	10.91	427.1970 425.1841	−1.40 2.49	3.2 × 10^7^ 1.8 × 10^7^
M7	Hydroxylation	C_22_H_26_N_4_O_4_	11.76	411.2023 409.1889	−0.93 1.88	7.7 × 10^7^ 1.3 × 10^6^
M8	Hydroxylation + *O*-Glucuronidation	C_28_H_34_N_4_O_10_	12.11	587.2347 585.2219	−0.12 2.88	3.4 × 10^6^ 2.9 × 10^6^
M9	*N*-Deethylation + Oxidation	C_20_H_20_N_4_O_4_	12.33	381.1552 ND	−1.39 -	3.5 × 10^6^ ND
M10	*N*-Deethylation + *N*-Deethylation	C_18_H_18_N_4_O_3_	12.81	339.1455 ND	0.98 -	2.2 × 10^7^ ND
M11	Hydroxylation + Oxidative deamination	C_18_H_17_N_3_O_5_	13.18	356.1245 ND	1.13 -	3.3 × 10^6^ ND
M12	Oxidation	C_22_H_24_N_4_O_4_	13.36	409.1876 ND	1.39 -	2.3 × 10^6^ ND
M13	*N*-Deethylation	C_20_H_22_N_4_O_3_	13.86	367.1759 ND	−1.54 -	5.1 × 10^8^ ND
Parent	Ethyleneoxynitazene	C_22_H_26_N_4_O_3_	15.16	395.2073 ND	−1.18 -	3.1 × 10^8^ ND
M14	*N*-Deethylation + Desaturation	C_20_H_20_N_4_O_3_	15.24	365.1608 ND	−0.05 -	1.6 × 10^7^ ND
M15	*N*-Deethylation + Oxidation	C_20_H_20_N_4_O_4_	16.91	381.1560 ND	0.70 -	1.2 × 10^7^ ND
M16	Oxidative deamination	C_18_H_17_N_3_O_4_	17.13	340.1298 ND	1.81 -	2.2 × 10^7^ ND

## Data Availability

Raw data were generated at the Department of Biomedical Sciences and Public Health at the Polytechnic University of Marche. Data derived from this study is available on request from the corresponding author.

## Supplementary Materials

The following supporting information can be downloaded at: https://www.mdpi.com/article/10.3390/metabo15100679/s1, Figure S1: High-resolution tandem mass spectrometry spectra after positive-electrospray ionization of ethyleneoxynitazene and metabolites using the data processing software FreeStyle (v.1.6, Thermo Scientific); Table S1: Inclusion list used during liquid chromatography-high-resolution tandem mass spectrometry (LC-HRMS/MS) for ethyleneoxynitazene identification; Table S2: Compound Discoverer processing settings for generating ethyleneoxynitazene putative metabolites; Table S3: Ethyleneoxynitazene putative metabolites predicted with BioTransformer v. 3.0 freeware.

## Abbreviations

The following abbreviations are used in this manuscript:

AGC	Automatic gain control
ddMS^2^	Data-dependent tandem mass spectrometry
FWHM	full width at half maximum
FullMS	Full-scan mass spectrometry
HESI	Heated electrospray ionization
HRMS/MS	High-resolution tandem mass spectrometry
IT	Injection time
LC	Liquid chromatography
MP	Mobile phase
NCE	Normalized collision energy
NSO	New synthetic opioid
RT	Retention time
SMILES	Simplified molecular-input line-entry system
sWME	Supplemented Williams’ medium E
