# Supplementary material for: In Vitro Metabolism of a Benzofuran-Substituted Nitazene: Ethyleneoxynitazene
Source: Metabolites. 2025 Oct 21;15(10):679. doi: 10.3390/metabo15100679 (PMC12566120; doi:10.3390/metabo15100679)
Supplement: Supplementary file 1 [file metabolites-15-00679-s001.zip › Taoussi_EthyleneoxyN_SuppFigS1_FINAL.pdf]

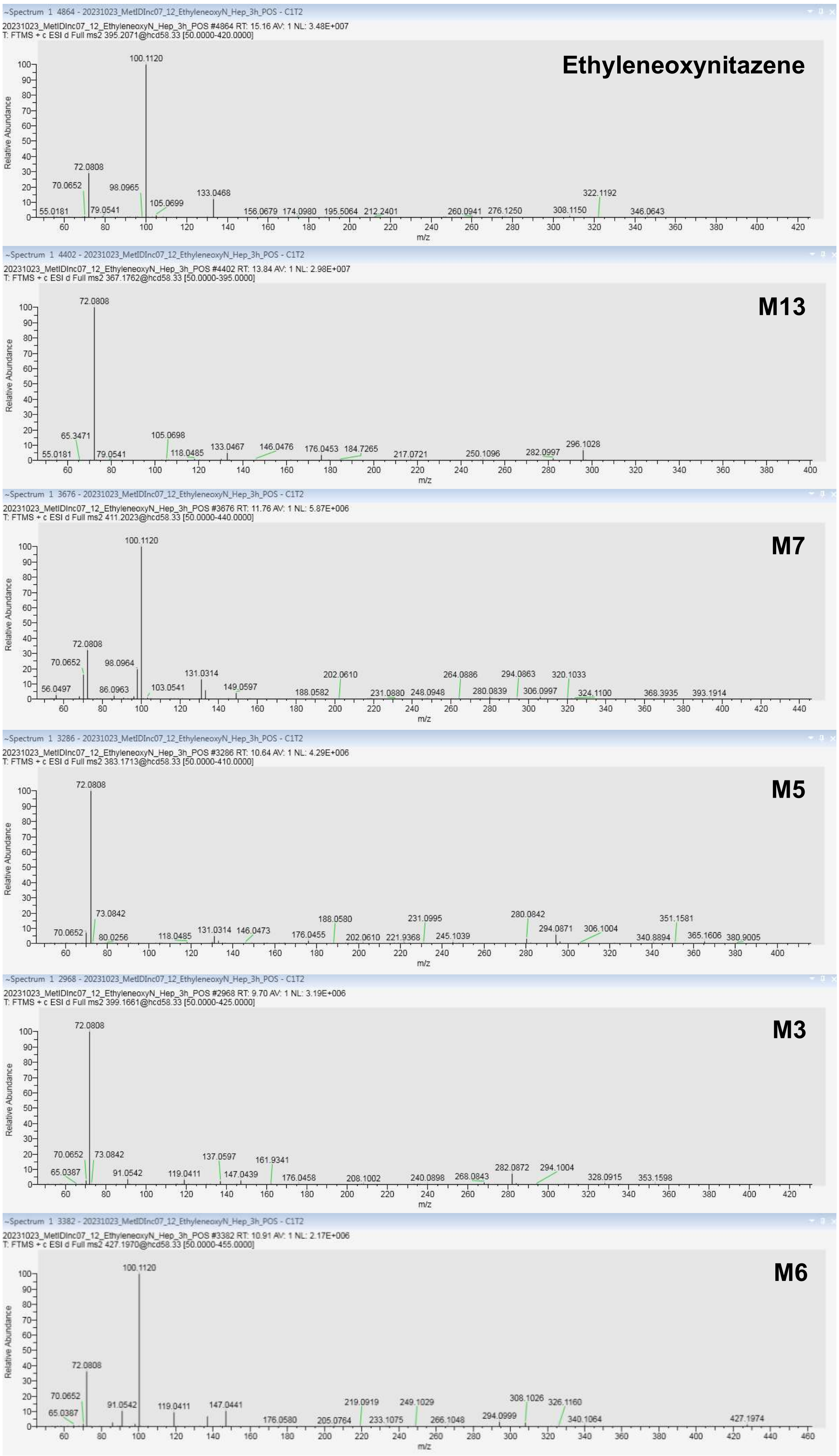

Figure S1. High-resolution tandem mass spectrometry spectra after positive-electrospray ionization of ethyleneoxynitazene and metabolites using the data processing software FreeStyle (v.1.6, Thermo Scientific).
