# Supplementary material for: In Vitro Metabolism of a Benzofuran-Substituted Nitazene: Ethyleneoxynitazene
Source: Metabolites. 2025 Oct 21;15(10):679. doi: 10.3390/metabo15100679 (PMC12566120; doi:10.3390/metabo15100679)
Supplement: Supplementary file 1 [file metabolites-15-00679-s001.zip › Taoussi_EthyleneoxyN_SuppTableS1_FINAL.pdf]

Table S1. Inclusion list used during liquid chromatography-high-resolution tandem mass spectrometry (LC-HRMS/MS) for ethyleneoxynitazene identification.

**Transformation**	**Elemental composition**	**$[M+H]^+$ $[M+H]^-$ *m/z***
Parent	$C_{22}H_{26}N_4O_3$	395.2078<br>393.1932
-2C-4H	$C_{20}H_{22}N_4O_3$	367.1765<br>365.1619
+2H+O	$C_{22}H_{28}N_4O_4$	413.2183<br>411.2038
+6C+10H+7O	$C_{28}H_{36}N_4O_{10}$	589.2504<br>587.2359
+O	$C_{22}H_{26}N_4O_4$	411.2027<br>409.1881
-2C-2H+O	$C_{20}H_{24}N_4O_4$	385.1870<br>383.1725
-4C-8H	$C_{18}H_{18}N_4O_3$	339.1452<br>337.1306
-2H+O	$C_{22}H_{24}N_4O_4$	409.1870<br>407.1725
-2C-4H+O	$C_{20}H_{22}N_4O_4$	383.1714<br>381.1568
+2H+2O	$C_{22}H_{28}N_4O_5$	429.2132<br>427.1987
+2O	$C_{22}H_{26}N_4O_5$	427.1976<br>425.1830
+6C+8H+7O	$C_{28}H_{34}N_4O_{10}$	587.2348<br>585.2202
+2H-2O	$C_{22}H_{28}N_4O$	365.2336<br>363.2190
-O	$C_{22}H_{26}N_4O_2$	379.2129<br>377.1983
+2H-O	$C_{22}H_{28}N_4O_2$	381.2285<br>379.2140
-2H+2O	$C_{22}H_{24}N_4O_5$	425.1819<br>423.1674
-4C-8H+O	$C_{18}H_{18}N_4O_4$	355.1401<br>353.1255
-2C-6H+O	$C_{20}H_{20}N_4O_4$	381.1557<br>379.1412

+2H+O	$C_{22}H_{28}N_4O_7S$	493.1751<br>491.1606
+4O+S	$C_{22}H_{26}N_4O_7S$	491.1595<br>489.1449
-4C-10H+O	$C_{18}H_{16}N_4O_4$	353.1244<br>351.1099
-4C-9H-N-O	$C_{18}H_{17}N_3O_4$	340.1292<br>338.1146
+2C+H+7O	$C_{24}H_{27}N_3O_{10}$	516.1613<br>514.1467
-4C-9H-N+2O	$C_{18}H_{17}N_3O_5$	356.1241<br>354.1095
-4C-9H-N+4O+S	$C_{18}H_{17}N_3O_7S$	420.0860<br>418.0714
