# Supplementary material for: In Vitro Metabolism of a Benzofuran-Substituted Nitazene: Ethyleneoxynitazene
Source: Metabolites. 2025 Oct 21;15(10):679. doi: 10.3390/metabo15100679 (PMC12566120; doi:10.3390/metabo15100679)
Supplement: Supplementary file 1 [file metabolites-15-00679-s001.zip › Taoussi_EthyleneoxyN_SuppTableS2_FINAL.pdf]

Table S2. Compound Discoverer processing settings for generating ethylneoxynitazene putative metabolites**.**

**Phase I reactions**	Dehydration (–2H -O → ø)<br>Desaturation (–2H → ø)<br>N-Deethylation (–2C -5H → +H)<br>Hydration (ø → +2H +O)<br>Ketone formation (–O → +2H)<br>Oxidation (ø → +O)<br>Oxidative deamination to alcohol (–2H –N → +H +O)<br>Oxidative deamination to ketone (–3H –N → +O)<br>Reduction (ø → 2H)
**Phase II reactions**	Acetylation (–H → +2C +3H +O)<br>Cysteine conjugation (–H → +3C +6H +N +2O +S)<br>Cysteine-Glycine conjugation (–H → +5C +9H +2N +3O +S)<br>Glucuronide conjugation (–H → +6C +9H +6O)<br>GSH conjugation (–H → +10C +15H +3N +6O +S)<br>Methylation (–H → +C +3H)<br>Sulfation (–H → +H +3O +S)
**Max number of dealkylations**	3
**Max number of phase II reactions**	2
**Max number of all steps**	5
**Adducts**	$[M+H]^+$<br>$[M-H]^-$
