# Supplementary material for: In Vitro Metabolism of a Benzofuran-Substituted Nitazene: Ethyleneoxynitazene
Source: Metabolites. 2025 Oct 21;15(10):679. doi: 10.3390/metabo15100679 (PMC12566120; doi:10.3390/metabo15100679)
Supplement: Supplementary file 1 [file metabolites-15-00679-s001.zip › Taoussi_EthyleneoxyN_SuppTableS3_FINAL.pdf]

Table S3. Ethyleneoxynitazene putative metabolites predicted with BioTransformer v. 3.0 freeware.

ID	Transformation	Elemental composition	Simplified molecular-input line-entry system (SMILES)
pA1	Hydroxylation	$C_{22}H_{26}N_4O_4$	O=[N+]([O-])C1=CC=C2N(CCN(CC)CC)C(=NC2=C1)C(C3=CC=C4OCCC4=C3)O
pA1-1	+ Oxidation of secondary alcohol to ketone	$C_{22}H_{24}N_4O_4$	O=[N+]([O-])C1=CC=C2N(CCN(CC)CC)C(=NC2=C1)C(C3=CC=C4OCCC4=C3)=O
pA1-2	+ Aromatic hydroxylation of fused benzene ring	$C_{22}H_{26}N_4O_5$	O=[N+]([O-])C1=C(C=C2N(CCN(CC)CC)C(=NC2=C1)C(C3=CC=C4OCCC4=C3)O)O
pA1-3	+ *N*-Oxidation of aliphatic tertiary amine	$C_{22}H_{26}N_4O_5$	O=[N+]([O-])C1=CC=C2N(CC[N+](CC)(CC)[O-])C(=NC2=C1)C(C3=CC=C4OCCC4=C3)O
pA1-4	+ Hydroxylation of heteroalicyclic secondary carbon	$C_{22}H_{26}N_4O_5$	O=[N+]([O-])C1=CC=C2N(CCN(CC)CC)C(=NC2=C1)C(C3=CC=C4OCC(C4=C3)O)O
pA1-5	+ Hydroxylation of terminal methyl	$C_{22}H_{26}N_4O_5$	O=[N+]([O-])C1=CC=C2N(CCN(CC)CCO)C(=NC2=C1)C(C3=CC=C4OCCC4=C3)O
pA1-6	+ *N*-Dealkylation of acyclic tertiary amine	$C_{20}H_{22}N_4O_4$	O=[N+]([O-])C1=CC=C2N(CCNCC)C(=NC2=C1)C(C3=CC=C4OCCC4=C3)O
pA1-7	+ Aliphatic hydroxylation of carbon alpha to secondary or tertiary alkyl-*N*	$C_{22}H_{26}N_4O_5$	O=[N+]([O-])C1=CC=C2N(CC(N(CC)CC)O)C(=NC2=C1)C(C3=CC=C4OCCC4=C3)O
pA1-8	+ Nitroreduction of nitroarene to hydroxylamine	$C_{22}H_{28}N_4O_3$	N(O)C1=CC=C2N(CCN(CC)CC)C(=NC2=C1)C(C3=CC=C4OCCC4=C3)O
pA1-9	+ Alkyl-OH-glucuronidation	$C_{28}H_{34}N_4O_{10}$	O=[N+]([O-])C1=CC=C2N(CCN(CC)CC)C(=NC2=C1)C(C3=CC=C4OCCC4=C3)OC5OC(C(O)=O)C(C(C5O)O)O
pA1-10	+ *N*-Glucuronidation of tertiary aliphatic amine	$[C_{28}H_{35}N_4O_{10}]^+$	O=[N+]([O-])C1=CC=C2N(CC[N+](CC)(CC)C3OC(C(O)=O)C(C(C3O)O)O)C(=NC2=C1)C(C4=CC=C5OCCC5=C4)O
pA1-11	+ Sulfation of secondary alcohol	$C_{22}H_{26}N_4O_7S$	O=[N+]([O-])C1=CC=C2N(CCN(CC)CC)C(=NC2=C1)C(C3=CC=C4OCCC4=C3)OS(O)(=O)=O
pA1-12	+ Nitroreduction of nitroarene to nitrosoarene	$C_{22}H_{26}N_4O_3$	O=NC1=CC=C2N(CCN(CC)CC)C(=NC2=C1)C(C3=CC=C4OCCC4=C3)O
pA2	Aromatic hydroxylation of fused benzene ring	$C_{22}H_{26}N_4O_4$	O=[N+]([O-])C1=C(C=C2N(CCN(CC)CC)C(=NC2=C1)CC3=CC=C4OCCC4=C3)O
pA2-1	+ Aromatic hydroxylation of fused benzene ring	$C_{22}H_{26}N_4O_5$	O=[N+]([O-])C1=C(C=C2N(CCN(CC)CC)C(=NC2=C1)CC3=C(C=C4OCCC4=C3)O)O
pA2-2	+ *N*-Oxidation of aliphatic tertiary amine	$C_{22}H_{26}N_4O_5$	O=[N+]([O-])C1=C(C=C2N(CC[N+](CC)(CC)[O-])C(=NC2=C1)CC3=CC=C4OCCC4=C3)O
pA2-3	+ Hydroxylation of heteroalicyclic secondary carbon	$C_{22}H_{26}N_4O_5$	O=[N+]([O-])C1=C(C=C2N(CCN(CC)CC)C(=NC2=C1)CC3=CC=C4OCC(C4=C3)O)O
pA2-4	+ Hydroxylation of terminal methyl	$C_{22}H_{26}N_4O_5$	O=[N+]([O-])C1=C(C=C2N(CCN(CC)CCO)C(=NC2=C1)CC3=CC=C4OCCC4=C3)O
pA2-5	+ N-Dealkylation of acyclic tertiary amine	$C_{18}H_{15}N_3O_5$	O=[N+]([O-])C1=C(C=C2N(CC=O)C(=NC2=C1)CC3=CC=C4OCCC4=C3)O
pA2-6	+ Nitroreduction	$C_{22}H_{28}N_4O_2$	C1(=C(C=C2N(CCN(CC)CC)C(=NC2=C1)CC3=CC=C4OCCC4=C3)O)N
pA2-7	+ Nitroreduction of nitroarene to hydroxylamine	$C_{22}H_{28}N_4O_3$	N(O)C1=C(C=C2N(CCN(CC)CC)C(=NC2=C1)CC3=CC=C4OCCC4=C3)O
pA2-8	+ Aromatic OH-glucuronidation	$C_{28}H_{34}N_4O_{10}$	O=[N+]([O-])C1=C(C=C2N(CCN(CC)CC)C(=NC2=C1)CC3=CC=C4OCCC4=C3)OC5OC(C(O)C(O)C5O)C(O)=O
pA2-9	+ *N*-Glucuronidation of tertiary aliphatic amine	$[C_{28}H_{35}N_4O_{10}]^+$	O=[N+]([O-])C1=C(C=C2N(CC[N+](CC)(CC)C3OC(C(O)=O)C(C(C3O)O)O)C(=NC2=C1)CC4=CC=C5OCCC5=C4)O
pA3	*N*-Oxidation of aliphatic tertiary amine	$C_{22}H_{26}N_4O_4$	O=[N+]([O-])C1=CC=C2N(CC[N+](CC)(CC)[O-])C(=NC2=C1)CC3=CC=C4OCCC4=C3
pA3-1	+ Hydroxylation of heteroalicyclic secondary carbon	$C_{22}H_{26}N_4O_5$	O=[N+]([O-])C1=CC=C2N(CC[N+](CC)(CC)[O-])C(=NC2=C1)CC3=CC=C4OCC(C4=C3)O
pA3-2	+ Aromatic hydroxylation of fused benzene ring	$C_{22}H_{26}N_4O_5$	O=[N+]([O-])C1=CC=C2N(CC[N+](CC)(CC)[O-])C(=NC2=C1)CC3=CC(=C4OCCC4=C3)O
pA3-3	+ Hydroxylation of terminal methyl	$C_{22}H_{26}N_4O_5$	O=[N+]([O-])C1=CC=C2N(CC[N+](CC)(CCO)[O-])C(=NC2=C1)CC3=CC=C4OCCC4=C3
pA3-4	+ *N*-Dealkylation	$[C_{20}H_{21}N_4O_4]^-$	O=[N+]([O-])C1=CC=C2N(CCN(CC)[O-])C(=NC2=C1)CC3=CC=C4OCCC4=C3
pA3-5	+ Hydroxylation	$C_{22}H_{26}N_4O_5$	O=[N+]([O-])C1=CC=C2N(CC[N+](CC)(CC)[O-])C(=NC2=C1)CC3=CC=C4OC(CC4=C3)O
pA3-6	+ Nitroreduction of nitroarene to hydroxylamine	$C_{22}H_{28}N_4O_3$	N(O)C1=CC=C2N(CC[N+](CC)(CC)[O-])C(=NC2=C1)CC3=CC=C4OCCC4=C3
pA3-7	+ *O*-Glucuronidation of *N*-oxide	$[C_{28}H_{35}N_4O_{10}]^+$	O=[N+]([O-])C1=CC=C2N(CC[N+](CC)(CC)OC3OC(C(O)C(O)C3O)C(O)=O)C(=NC2=C1)CC4=CC=C5OCCC5=C4
pA3-8	Nitroreduction of nitroarene to nitrosoarene	$C_{22}H_{26}N_4O_3$	O=NC1=CC=C2N(CC[N+](CC)(CC)[O-])C(=NC2=C1)CC3=CC=C4OCCC4=C3
pA4	Hydroxylation of heteroalicyclic secondary carbon	$C_{22}H_{26}N_4O_4$	O=[N+]([O-])C1=CC=C2N(CCN(CC)CC)C(=NC2=C1)CC3=CC=C4OCC(C4=C3)O
pA4-1	+ Oxidation of secondary alcohol to ketone	$C_{22}H_{24}N_4O_4$	O=[N+]([O-])C1=CC=C2N(CCN(CC)CC)C(=NC2=C1)CC3=CC=C4OCC(C4=C3)=O
pA4-2	+ Aromatic hydroxylation of fused benzene ring	$C_{22}H_{26}N_4O_5$	O=[N+]([O-])C1=CC=C2N(CCN(CC)CC)C(=NC2=C1)CC3=CC(=C4OCC(C4=C3)O)O
pA4-3	+ Hydroxylation of terminal methyl	$C_{22}H_{26}N_4O_5$	O=[N+]([O-])C1=CC=C2N(CCN(CC)CCO)C(=NC2=C1)CC3=CC=C4OCC(C4=C3)O
pA4-4	+ N-Dealkylation of acyclic tertiary amine	$C_{20}H_{22}N_4O_4$	O=[N+]([O-])C1=CC=C2N(CCNCC)C(=NC2=C1)CC3=CC=C4OCC(C4=C3)O
pA4-5	+ Aliphatic hydroxylation of carbon alpha to secondary or tertiary alkyl-*N*	$C_{22}H_{26}N_4O_5$	O=[N+]([O-])C1=CC=C2N(CC(N(CC)CC)O)C(=NC2=C1)CC3=CC=C4OCC(C4=C3)O
pA4-6	+ Nitroreduction of nitroarene to hydroxylamine	$C_{22}H_{28}N_4O_3$	N(O)C1=CC=C2N(CCN(CC)CC)C(=NC2=C1)CC3=CC=C4OCC(C4=C3)O
pA4-7	+ Alkyl-OH-glucuronidation	$C_{28}H_{34}N_4O_{10}$	O=[N+]([O-])C1=CC=C2N(CCN(CC)CC)C(=NC2=C1)CC3=CC=C4OCC(C4=C3)OC5OC(C(O)=O)C(C(C5O)O)O
pA4-8	+ *N*-Glucuronidation of tertiary aliphatic amine	$[C_{28}H_{35}N_4O_{10}]^+$	O=[N+]([O-])C1=CC=C2N(CC[N+](CC)(CC)C3OC(C(O)=O)C(C(C3O)O)O)C(=NC2=C1)CC4=CC=C5OCC(C5=C4)O
pA4-9	+ Sulfation	$C_{22}H_{26}N_4O_7S$	O=[N+]([O-])C1=CC=C2N(CCN(CC)CC)C(=NC2=C1)CC3=CC=C4OCC(C4=C3)OS(O)(=O)=O
pA4-10	+ Nitroreduction of nitroarene to nitrosoarene	$C_{22}H_{26}N_4O_3$	O=NC1=CC=C2N(CCN(CC)CC)C(=NC2=C1)CC3=CC=C4OCC(C4=C3)O
pA5	Hydroxylation of terminal methyl	$C_{22}H_{26}N_4O_4$	O=[N+]([O-])C1=CC=C2N(CCN(CC)CCO)C(=NC2=C1)CC3=CC=C4OCCC4=C3
pA5-1	+ Oxidation of primary alcohol to aldehyde	$C_{22}H_{24}N_4O_4$	O=[N+]([O-])C1=CC=C2N(CCN(CC)CC=O)C(=NC2=C1)CC3=CC=C4OCCC4=C3

pA5-2	+ Hydroxylation of terminal methyl	$C_{22}H_{26}N_4O_5$	O=[N+]([O-])C1=CC=C2N(CCN(CCO)CCO)C(=NC2=C1)CC3=CC=C4OCCC4=C3
pA5-3	+ *N*-Dealkylation of acyclic tertiary amine	$C_{20}H_{22}N_4O_4$	O=[N+]([O-])C1=CC=C2N(CCNCCO)C(=NC2=C1)CC3=CC=C4OCCC4=C3
pA5-4	+ Aliphatic hydroxylation of carbon alpha to secondary or tertiary alkyl-*N*	$C_{22}H_{26}N_4O_5$	O=[N+]([O-])C1=CC=C2N(CCN(C(C)O)CCO)C(=NC2=C1)CC3=CC=C4OCCC4=C3
pA5-5	+ Hydroxylation	$C_{22}H_{26}N_4O_5$	O=[N+]([O-])C1=CC=C2N(CCN(CC)CCO)C(=NC2=C1)CC3=CC=C4OC(CC4=C3)O
pA5-6	+ Nitroreduction of nitroarene to hydroxylamine	$C_{22}H_{28}N_4O_3$	N(O)C1=CC=C2N(CCN(CC)CCO)C(=NC2=C1)CC3=CC=C4OCCC4=C3
pA5-7	+ Alkyl-OH-glucuronidation	$C_{28}H_{34}N_4O_{10}$	O=[N+]([O-])C1=CC=C2N(CCN(CC)CCOC3OC(C(O)=O)C(C(C3O)O)O)C(=NC2=C1)CC4=CC=C5OCCC5=C4
pA5-8	+ *N*-Glucuronidation of tertiary aliphatic amine	$[C_{28}H_{35}N_4O_{10}]^+$	O=[N+]([O-])C1=CC=C2N(CC[N+](CC)(CCO)C3OC(C(O)=O)C(C(C3O)O)O)C(=NC2=C1)CC4=CC=C5OCCC5=C4
pA5-9	+ Sulfation	$C_{22}H_{26}N_4O_7S$	O=[N+]([O-])C1=CC=C2N(CCN(CC)CCOS(O)(=O)=O)C(=NC2=C1)CC3=CC=C4OCCC4=C3
pA5-10	+ Nitroreduction of nitroarene to nitrosoarene	$C_{22}H_{26}N_4O_3$	O=NC1=CC=C2N(CCN(CC)CCO)C(=NC2=C1)CC3=CC=C4OCCC4=C3
pA6	*N*-Dealkylation of acyclic tertiary amine	$C_{20}H_{22}N_4O_3$	O=[N+]([O-])C1=CC=C2N(CCNCC)C(=NC2=C1)CC3=CC=C4OCCC4=C3
pA6-1	+ *N*-Dealkylation of acyclic secondary amine	$C_{18}H_{15}N_3O_4$	O=[N+]([O-])C1=CC=C2N(CC=O)C(=NC2=C1)CC3=CC=C4OCCC4=C3
pA6-2	+ *N*-Dealkylation of acyclic secondary amine	$C_{18}H_{18}N_4O_3$	O=[N+]([O-])C1=CC=C2N(CCN)C(=NC2=C1)CC3=CC=C4OCCC4=C3
pA6-3	+ Hydroxylation	$C_{20}H_{22}N_4O_4$	O=[N+]([O-])C1=CC=C2N(CCNCC)C(=NC2=C1)CC3=CC=C4OC(CC4=C3)O
pA6-4	+ Nitroreduction of nitroarene to hydroxylamine	$C_{20}H_{24}N_4O_2$	N(O)C1=CC=C2N(CCNCC)C(=NC2=C1)CC3=CC=C4OCCC4=C3
pA6-5	+ Nitroreduction of nitroarene to nitrosoarene	$C_{20}H_{22}N_4O_2$	O=NC1=CC=C2N(CCNCC)C(=NC2=C1)CC3=CC=C4OCCC4=C3
pA7	Aliphatic hydroxylation of carbon alpha to secondary or tertiary alkyl-*N*	$C_{22}H_{26}N_4O_4$	O=[N+]([O-])C1=CC=C2N(CC(N(CC)CC)O)C(=NC2=C1)CC3=CC=C4OCCC4=C3
pA7-1	+ *N*-Oxidation of aliphatic tertiary amine	$C_{22}H_{26}N_4O_5$	O=[N+]([O-])C1=CC=C2N(CC([N+](CC)(CC)[O-])O)C(=NC2=C1)CC3=CC=C4OCCC4=C3
pA7-2	+ *N*-Dealkylation of acyclic tertiary amine	$C_{18}H_{15}N_3O_5$	O=[N+]([O-])C1=CC=C2N(CC(=O)O)C(=NC2=C1)CC3=CC=C4OCCC4=C3
pA7-3	+ *N*-Dealkylation of acyclic tertiary amine	$C_{20}H_{22}N_4O_4$	O=[N+]([O-])C1=CC=C2N(CC(NCC)O)C(=NC2=C1)CC3=CC=C4OCCC4=C3
pA7-4	+ Hydroxylation	$C_{22}H_{26}N_4O_5$	O=[N+]([O-])C1=CC=C2N(CC(N(CC)CC)O)C(=NC2=C1)CC3=CC=C4OC(CC4=C3)O
pA7-5	+ Nitroreduction of nitroarene to hydroxylamine	$C_{22}H_{28}N_4O_3$	N(O)C1=CC=C2N(CC(N(CC)CC)O)C(=NC2=C1)CC3=CC=C4OCCC4=C3
pA7-6	+ *N*-Glucuronidation of tertiary aliphatic amine	$[C_{28}H_{35}N_4O_{10}]^+$	O=[N+]([O-])C1=CC=C2N(CC([N+](CC)(CC)C3OC(C(O)=O)C(C(C3O)O)O)O)C(=NC2=C1)CC4=CC=C5OCCC5=C4
pA7-7	+ Nitroreduction of nitroarene to nitrosoarene	$C_{22}H_{26}N_4O_3$	O=NC1=CC=C2N(CC(N(CC)CC)O)C(=NC2=C1)CC3=CC=C4OCCC4=C3
pA8	Hydroxylation	$C_{22}H_{26}N_4O_4$	O=[N+]([O-])C1=CC=C2N(CCN(CC)CC)C(=NC2=C1)CC3=CC=C4OC(CC4=C3)O
pA8-1	+ Hydroxylation	$C_{22}H_{26}N_4O_5$	O=[N+]([O-])C1=CC=C2N(CCN(CC)CC)C(=NC2=C1)CC3=CC=C4OC(C(C4=C3)O)O
pA8-2	+ Nitroreduction of nitroarene to hydroxylamine	$C_{22}H_{28}N_4O_3$	N(O)C1=CC=C2N(CCN(CC)CC)C(=NC2=C1)CC3=CC=C4OC(CC4=C3)O
pA8-3	+ *N*-Glucuronidation of tertiary aliphatic amine	$[C_{28}H_{35}N_4O_{10}]^+$	O=[N+]([O-])C1=CC=C2N(CC[N+](CC)(CC)C3OC(C(O)=O)C(C(C3O)O)O)C(=NC2=C1)CC4=CC=C5OC(CC5=C4)O
pA8-4	+ Nitroreduction of nitroarene to nitrosoarene	$C_{22}H_{26}N_4O_3$	O=NC1=CC=C2N(CCN(CC)CC)C(=NC2=C1)CC3=CC=C4OC(CC4=C3)O
pA9	*N*-Glucuronidation of tertiary aliphatic amine	$[C_{28}H_{35}N_4O_9]^+$	O=[N+]([O-])C1=CC=C2N(CC[N+](CC)(CC)C3OC(C(O)=O)C(C(C3O)O)O)C(=NC2=C1)CC4=CC=C5OCCC5=C4
pA10	Nitroreduction of nitroarene to hydroxylamine	$C_{22}H_{28}N_4O_2$	N(O)C1=CC=C2N(CCN(CC)CC)C(=NC2=C1)CC3=CC=C4OCCC4=C3
pA10-1	+ *O*-Acetylation of *N*-hydroxylarylamine	$C_{24}H_{30}N_4O_3$	N(OC(C)=O)C1=CC=C2N(CCN(CC)CC)C(=NC2=C1)CC3=CC=C4OCCC4=C3
pA10-2	+ Reduction of arylhydroxylamine to arylamine	$C_{22}H_{28}N_4O$	NC1=CC=C2N(CCN(CC)CC)C(=NC2=C1)CC3=CC=C4OCCC4=C3
pA10-3	+ *N*-Glucuronidation of hydroxylamine	$C_{28}H_{36}N_4O_8$	N(O)(C1=CC=C2N(CCN(CC)CC)C(=NC2=C1)CC3=CC=C4OCCC4=C3)C5OC(C(O)C(O)C5O)C(O)=O
pA10-4	+ *N*-Glucuronidation of tertiary aliphatic amine	$[C_{28}H_{37}N_4O_8]^+$	N(O)C1=CC=C2N(CC[N+](CC)(CC)C3OC(C(O)=O)C(C(C3O)O)O)C(=NC2=C1)CC4=CC=C5OCCC5=C4
pA10-5	+ Oxidation	$C_{22}H_{28}N_4O_3$	O[NH+](C1=CC=C2N(CCN(CC)CC)C(=NC2=C1)CC3=CC=C4OCCC4=C3)[O-]
pA11	Nitroreduction of nitroarene to nitrosoarene	$C_{22}H_{26}N_4O_2$	O=NC1=CC=C2N(CCN(CC)CC)C(=NC2=C1)CC3=CC=C4OCCC4=C3
pA11-1	+ *N*-Glucuronidation of tertiary aliphatic amine	$[C_{28}H_{35}N_4O_8]^+$	O=NC1=CC=C2N(CC[N+](CC)(CC)C3OC(C(O)=O)C(C(C3O)O)O)C(=NC2=C1)CC4=CC=C5OCCC5=C4
